# The global activity limitation indicator and self-rated health: two complementary predictors of mortality

**DOI:** 10.1186/s13690-015-0073-0

**Published:** 2015-05-11

**Authors:** Nicolas Berger, Johan Van der Heyden, Herman Van Oyen

**Affiliations:** Department of Social & Environmental Health Research, London School of Hygiene & Tropical Medicine, London, UK; Public Health and Surveillance, Scientific Institute of Public Health, Rue Juliette Wytsmanstraat 14, 1050 Brussels, Belgium; Department of Public Health, Ghent University, Ghent, Belgium

**Keywords:** GALI, Self-rated health, Global indicator, Disability, Socioeconomic status, Mortality, Predictive ability, Healthy Life Years, Health Expectancy

## Abstract

**Background:**

The purpose of this study is to compare the ability of the Global Activity Limitation Indicator (GALI) and self-rated health (SRH) to predict all-cause mortality in the general adult population.

**Methods:**

We linked the 2001 Belgian Health Interview Survey with mortality and migration registers 2001–2010. The baseline sample included 8,583 individuals aged 15 years and older. Poisson regression models were used to estimate the effect of the GALI and SRH on mortality rate during follow-up. We investigated the impact of gender, age, education and follow-up period on the association between the GALI/SRH and mortality.

**Results:**

The GALI and SRH were strong and complementary predictors of mortality in the Belgian adult population. Although the two global instruments shared some traits, they predicted mortality concurrently, with some indication of a somewhat stronger effect for SRH. We found neither significant differences between men and women, nor between education groups. The predictive effect of the GALI and SRH slightly decreased over time and the predictive effect of SRH slightly decreased with age.

**Conclusions:**

Our findings suggest that the GALI and SRH are useful and complementary measures for assessing the health and functional status of adults in population surveys.

## Background

The Global Activity Limitation Indicator (GALI) refers to a single-item measure of functional status where individuals are asked to rate their long-term limitations in usual activities due to a health problem [1-3]. This comprehensive instrument is the underlying measure of the European indicator ‘Healthy Life Years’ and is used together with self-rated health (SRH) and a chronic morbidity question – the set of 3 questions defines the Minimum European Health Module [2] – to monitor population health and functioning in the European Union [4-7]. Despite its simplicity, SRH is a powerful instrument as it has been shown to be highly predictive for mortality in different subgroups of the population and in different cultures [8-12]. Attempts have been made to understand whether the subjective assessment of health status is associated with mortality in a similar manner in different population subgroups, with lately particular attention on the moderating influence of socioeconomic status. Whereas the predictive ability of SRH for mortality appears to weaken for females in most settings, studies investigating the moderating influence of socioeconomic status have shown mixed results, leaving uncertainty about the measurement equivalence of the instrument across population subgroups [11,13-17].

As opposed to SRH, the predictive power of the GALI on mortality remains relatively unexplored. Since the GALI was first used to calculate the European indicator ‘Healthy Life Years’ in 2004 [5], several national and cross-national studies have shown its content validity, showing that the global item correlates with detailed instruments of functioning and disability in a similar way across Europe [3,18-20]. More recently, several studies suggested that both the GALI and SRH are good predictors of mortality in Belgium and Europe [21,22]. The predictive ability of the two items partly overlapped in these studies; yet each measure had its own impact on mortality. These results were expected because the GALI and SRH aim to capture separate dimensions: the GALI mainly measures functioning while SRH focusses on health status [19]. The studies did not allow to determine whether gender, age, and/or socioeconomic status modify the effect of the GALI on mortality. They reached opposed conclusions regarding the role of gender, whereas the influence of age and socioeconomic status have not been explored to date. Current findings are also restricted to short follow-up periods (2–3 years) and to older populations (50+ and 65+). It is therefore unclear whether the GALI has a predictive power on long-term mortality and in the larger adult population. Noteworthy data shortcomings – substantial attrition problems in the first study [22] and the absence of the exact date of death in the second [21] – further restrict the generalisability of these first findings.

The objective of our study is to investigate and compare the predictive ability of the GALI and SRH on mortality in the Belgian adult population combining a national survey of the adult population (15+) with a long period of mortality follow-up (10 years). We expect the two measures to complement each other to predict mortality because the GALI and SRH focus on different health aspects. To be a useful global instrument, the GALI should have an added predictive value over SRH.

Our study also aims to investigate how gender, age and socioeconomic status moderate the relationship between mortality and both global measures, and to evaluate whether the length of follow-up affects these relationships.

## Methods

### Data

Data are drawn from the 2001 Belgian Health Interview Survey (HIS). The 2001 HIS is a national survey in which 12,111 individuals living in Belgium were interviewed [23]. The survey was carried out by Statistics Belgium and exempt from ethics approval by law. Participants were selected from the National Register using a multistage sampling procedure. Household response rate was 61.3%. The GALI and SRH questions were part of the self-administered questionnaire restricted to individuals aged 15 years and older. Vital and migration status of the participants were followed up: records of the survey were linked to the National Register using a unique identifier present in both data sources. After the approval of the Belgian Commission for the protection of privacy, we obtained a mortality and emigration follow-up of HIS participants until 31 December 2010 (approximately 10 years). The linkage was successful for 97% of the records. The final HIS sample included 8,583 individuals aged 15 years and older, of whom 902 died and 132 emigrated. Emigrants were considered as lost to follow-up at the date of emigration.

### Measures

Age was obtained from the National Register and was categorised into four groups: persons aged 15–49 (reference category), persons aged 50–64, persons aged 65–74 and persons aged 75 or more. Given their low probability of death, participants aged 15–49 were grouped in a single category. Sensitivity analysis ensured that the age group cut-offs did not affect the results. Other variables, apart from mortality, were drawn from the baseline questionnaire in 2001. Education was measured using the highest education level achieved within the household. We recoded this measure into four categories: primary education level or lower, lower secondary, higher secondary, and higher education [24].

Activity limitation was assessed with the GALI question: ‘For the past 6 months or more, have you been limited in activities people usually do because of a health problem? Yes, strongly limited/Yes, limited/No, not limited’ [2]. Within the ICF framework [25], the GALI corresponds to the societal perspective of functioning, i.e. the performance of roles and social involvement in activities. The reference to ‘activities people usually do’ fosters normative comparisons and allows to account for differences in performance of roles by age, gender, environmental and living conditions. SRH was measured on a five-point scale, based on the question: ‘How is your health in general?’ [26]. To ease comparison with the GALI, we recoded SRH into three categories: (very) bad, fair, and (very) good.

### Statistical methods

We used Poisson regression models to estimate the effect of the GALI and SRH on the mortality rate during the follow-up period [27]. The first model (Model 1) estimates Mortality Rates Ratios (MRRs) for sociodemographic variables alone. Model 2 and Model 3 estimate MRRs for the GALI and SRH separately, while adjusting for sociodemographic factors. Variants of these models additionally estimate interaction effects between gender, age and education and each measure of ill health (results not reproduced). The full model (Model 4) includes all predictors (without interactions), allowing to compare the relative predictive ability of the GALI and SRH. For significant moderators, stratum-specific estimates of the GALI and SRH are presented for each value of the sociodemographic moderator. The impact of the follow-up period was assessed by comparing MRRs at different times of the follow-up, adjusting for all covariates. We did not use other measures of health and functioning to adjust the relationship between the GALI or SRH and mortality because we aimed to assess the importance of the GALI compared to SRH as predictors of mortality. Adjusting for other health and functioning measures would affect the GALI and SRH unequally and prevent rigorous comparison between the two global instruments [19]. All analyses were conducted using Stata 12 (StataCorp, College Station, USA).

## Results

22.20% of respondents reported a long-term activity limitation and 25.04% reported a fair or bad health at the interview (Table 1). Men, respondents with a lower education level, and older individuals had a higher mortality rate. Mortality rate also increased as the level of ill health increased: mortality rate was about 4 times higher (=26.38/6.31) for individuals with moderate activity limitations and more than 9 times higher (=60.02/6.31) for those with severe limitations, as compared to individuals without limitations. For SRH, mortality rate ratios (MRRs) were very comparable to those of the GALI (i.e. 3.93 (=24.40/6.21) and 9.90 (=61.49/6.21), for those reporting SRH as fair or (very) bad respectively).

**Table 1:** **Characteristics of the 2001 Belgian Health Interview Survey sample and mortality rate (per 1,000 person-years) during follow-up until 31/12/2010**

		**Distribution in sample**	**Deaths (N)**	**Mortality rate**
**Age**				
	15-49	58.28	72	1.54
	50-64	21.88	159	9.37
	65-74	11.81	262	31.01
	75 or higher	8.03	409	88.37
**Gender**				
	Men	48.81	508	13.72
	Women	51.19	394	9.90
**Education**				
	Primary	15.54	342	30.94
	Lower secondary	19.11	222	15.27
	Higher secondary	30.32	196	8.24
	Tertiary	35.03	142	5.17
**Activity limitations (GALI)**				
	No limitation	77.80	387	6.31
	Moderate limitation	17.15	327	26.38
	Severe limitation	5.04	188	60.02
**Self-rated health (SRH)**				
	(Very) good	74.96	367	6.21
	Fair	20.62	367	24.40
	(Very) bad	4.42	168	61.49

When adjusting for sociodemographic predictors of mortality (age, gender and education), MRRs decreased for both the GALI and SRH, but remained significant (Table 2). In a global model including the GALI, SRH and other covariates (Model 4), both measures had a significant effect on mortality. Moderate activity limitations and fair health both increased mortality rates by almost 1.5. The effect of severe limitations was slightly higher (MRR = 1.79) whereas the effect of (very) bad health was the strongest (MRR = 2.50). Although the crude MRRs were similar for the GALI and SRH, the predictive power of very bad SRH on mortality was slightly higher after adjustment.

**Table 2:** Mortality rate ratios* (and 95% CI) of the GALI, SRH, gender and education during 10-year follow-up, 2001 Belgian Health Interview Survey

		**Model 1**	**Model 2**	**Model 3**	**Model 4**
		**(Sociodemographics)**	**(Sociodemographics + GALI)**	**(Sociodemographics + SRH)**	**(Sociodemographics + GALI + SRH)**
**Age**					
	15-49 (ref cat)	1.00	1.00	1.00	1.00
	50-64	5.61	5.11	5.02	4.92
		(4.24-7.41)	(3.86-6.76)	(3.79-6.64)	(3.79-6.52)
	65-74	17.21	14.19	14.22	13.54
		(13.20-22.45)	(10.84-18.58)	(10.86-18.61)	(10.34-17.75)
	75 or higher	49.80	37.23	38.84	35.72
		(38.51-64.40)	(28.59-48.49)	(29.89-50.46)	(27.42-46.55)
**Gender**					
	Men (ref cat)	1.00	1.00	1.00	1.00
	Women	0.57	0.55	0.54	0.54
		(0.50-0.65)	(0.48-0.63)	(0.47-0.61)	(0.47-0.61)
**Education**					
	Primary	1.98	1.74	1.63	1.61
		(1.62-2.43)	(1.42-2.13)	(1.33-2.01)	(1.32-1.98)
	Lower secondary	1.59	1.49	1.43	1.43
		(1.29-1.97)	(1.21-1.85)	(1.16-1.78)	(1.15-1.77)
	Higher secondary	1.31	1.32	1.25	1.28
		(1.05-1.62)	(1.06-1.61)	(1.01-1.56)	(1.03-1.59)
	Tertiary (ref cat)	1.00	1.00	1.00	1.00
**Activity limitations (GALI)**					
	No limitation (ref cat)		1.00		1.00
	Moderate limitation		1.77		1.37
			(1.52-2.06)		(1.16-1.63)
	Severe limitation		2.96		1.79
			(2.46-3.56)		(1.42-2.25)
**Self-rated health (SRH)**					
	(Very) good (ref cat)			1.00	1.00
	Fair			1.78	1.49
				(1.53-2.07)	(1.26-1.77)
	(Very) bad			3.63	2.50
				(3.00-4.39)	(1.97-3.18)
**log likelihood**		−2711.73	−2644.65	−2629.60	−2616.87

*: from a Poisson regression model.

CI Confidence interval; GALI global activity limitation indicator; SRH self-rated health.

The predictive ability of the GALI and SRH for mortality did not vary with gender or with education (results not shown). Yet, age moderated the effect of SRH on mortality (significant interaction). As age increased, the predictive ability on mortality of both fair and (very) bad health decreased (Table 3). The MRR of fair health decreased from 2.89 to 1.24 from the youngest age group (15–49) to the oldest (75+) and the MRR of (very) bad health decreased from 4.82 to 1.74. Despite this trend, there is evidence that SRH predicted mortality in all age groups (with weaker evidence for fair health in population older than 75 years). Age did not affect the GALI in the same manner. Even though age-specific effects of the GALI on mortality slightly varied (Table 3), no clear pattern emerged and there was no significant interaction between age and the GALI. There was not enough evidence for a significant effect of the GALI in the youngest age group (due to the modest estimated effects and the low number of deaths). Moderate limitations in particular seemed to have little or no impact on mortality in individuals younger than 65 years. Comparison between the GALI and SRH indicates that the mortality impact of the GALI seemed lower in the younger age group and of similar magnitude in the older age group, as compared to SRH.

**Table 3:** Mortality rate ratios* (and 95% CI) of the GALI and SRH by age group, 2001 Belgian Health Interview Survey

		**15-49**		**50-64**		**65-74**		**75+**	
**Activity limitations (GALI)**									
	No limitation (ref cat)	1.00		1.00		1.00		1.00	
	Moderate limitation	1.00	(0.48-2.06)	1.25	(0.82-1.92)	1.42	(1.03-1.96)	1.33	(1.04-1.69)
	Severe limitation	2.00	(0.77-5.21)	2.52	(1.44-4.40)	1.77	(1.16-2.70)	1.69	(1.22-2.33)
**Self-rated health (SRH)**									
	(Very) good (ref cat)	1.00		1.00		1.00		1.00	
	Fair	2.89	(1.58-5.26)	1.93	(1.28-2.92)	1.38	(1.01-1.90)	1.24	(0.97-1.58)
	(Very) bad	4.82	(1.77-13.12)	4.03	(2.24-7.25)	2.80	(1.82-4.30)	1.74	(1.23-2.45)

*: from a Poisson regression model adjusted for gender, education and the other health measure.

CI Confidence interval; GALI global activity limitation indicator; SRH self-rated health.

The length of follow-up did not appear to play a large role in the findings. We estimated models that restricted the time of follow-up to 0–3 years, 3–6 years and 6–10 years and found significant effects on mortality in all periods (p < 0.05) (Table 4). Yet, overall the impacts of the GALI and SRH decreased over time. For instance, the effect of severe limitations decreased from 2.82 (in the first 3 years of follow-up) to 1.49 (in the last period). The effect of moderate limitations and fair health evolved in a similar fashion with a slight decrease in the MRR as the length of follow-up increased. It appears however that the MRR of (very) bad health was less affected by the time of follow-up. The MRR dropped from 3.32 to 2.20 midway through follow-up and slightly increased to 2.62 in the last period of follow-up. Although the MRRs of having severe limitations and being in (very) bad health were higher compared to the MRRs of having moderate limitations and being in fair health, respectively, the difference was only statistically significant for SRH in the last period of follow-up.

**Table 4:** Mortality rate ratios* (and 95% CI) of the GALI and SRH by period of follow-up, 2001 Belgian Health Interview Survey

		**0-3 years**		**3-6 years**		**6-10 years**	
**Activity limitations (GALI)**							
	No limitation (ref cat)	1.00		1.00		1.00	
	Moderate limitation	1.56	(1.05-2.33)	1.46	(1.08-1.97)	1.29	(1.00-1.66)
	Severe limitation	2.82	(1.77-4.50)	1.70	(1.13-2.56)	1.49	(1.04-2.13)
**Self-rated health (SRH)**							
	(Very) good (ref cat)	1.00		1.00		1.00	
	Fair	1.82	(1.23-2.70)	1.69	(1.26-2.26)	1.29	(1.01-1.65)
	(Very) bad	3.32	(2.03-5.42)	2.20	(1.43-3.39)	2.62	(1.81-3.78)

*: from a Poisson regression model adjusted for age, gender, education and the other health measure.

CI Confidence interval; GALI global activity limitation indicator; SRH self-rated health.

## Discussion

This study shows that the Global Activity Limitation Indicator and self-rated health are strong and complementary predictors of mortality in the Belgian adult population. Although the two indicators share some characteristics, they predict mortality concurrently, with some indications of a somewhat stronger effect for SRH. There were neither significant differences between men and women, nor between education groups. The predictive ability of SRH appeared to slightly decrease with age. The effect of the global measures on mortality slightly decreased over time; yet we found that moderate limitations and fair health still allowed to predict mortality after more than 6 years of follow-up. Severe limitations had a slightly stronger effect, while the effect of (very) bad health remained strong towards the end of the follow-up.

Using **–** for the first time **–** a long (10 years) and accurate (97% of successful linkage) mortality follow-up we showed that the GALI is a complementary predictor of mortality to SRH in the whole adult population of Belgium. Two previous studies that examined this issue have reported an independent effect of the GALI on mortality in the older population in Europe and Belgium, respectively. The first study used the Survey of Health, Ageing and Retirement in Europe (SHARE) to estimate the mortality impact of the GALI and SRH in the presence of other health covariates [22]. The design of that study did not allow to compare the predictive power of the two global indicators because other covariates of the model overlapped the health dimensions captured by the GALI and SRH unequally. Yet, the study concluded that the GALI was a strong predictor of mortality which was, to a great extent, independent of SRH. The second study directly compared the GALI and SRH and concluded that the GALI was as strong as SRH (and stronger in women) for predicting mortality in the Belgian older population [21]. In the current analysis we found that SRH had a slightly higher predictive power (although there was no significant difference between GALI and SRH estimates), which decreased with age. Our results indicate that in the older population, the GALI and SRH seem to have similar predictive ability for mortality, which partly corroborates the earlier study.

The fact that we did not find gender differences in the predictive ability of the GALI for mortality is consistent with the Belgian study of the older population mentioned above [21]. In the SHARE study, the model excluding other health covariates also found similar estimates for males and females, although estimates were significant for women only in the model including additional health variables [22]. In a similar fashion, we found no gender differences for the effect of SRH on mortality, which is consistent with studies carried out in Sweden [13] and the United Kingdom [28].

This study also explored whether education modified the association between the GALI and mortality. The predictive powers of the GALI and SRH were consistent across education groups. This corroborates a Spanish study which showed that the GALI was neither influenced by gender nor by social class, after controlling for other functional status variables [19]. Results are also in line with Swedish and English studies that found no moderating effect of socioeconomic status in the relationship between SRH and mortality [13,16].

Overall, the associations between the two global measures and mortality were invariant to sociodemographic variables included in our study, with the exception of age for SRH. Such results suggest that the GALI, and to a lower extent SRH, provide comparable information across population subgroups. This reinforces the relevance of global measures of health for assessing health and functioning. Further research is needed to confirm our findings in different settings. Further research should also investigate the answering behaviour for the GALI question and the processes by which respondents adapt their answers depending on their sociodemographic and cultural settings. It would be particularly relevant to understand to what extent these processes differ between the GALI and SRH with respect to age.

Our analysis has several strengths: we were able to use one large national survey which contained health and mortality data; more than 97% of the survey participants could be linked to the National Register to obtain the exact date of death and emigration (loss to follow-up); and the length of the follow-up (10 years) allowed to differentiate short-term from long-term effects of the GALI and SRH on mortality.

There is an important limitation related to the findings reported here. The response rate of the Belgian Health Interview Survey (61.30%) and the underrepresentation of institutionalised populations may bias the results [29]. Previous studies showed that participation in the survey depends on health status and socioeconomic position [30,31]. Mortality rates based on survey follow-up tend to be underestimated, but not to the same extent across all education groups. A recent study showed that lowly educated participants in the 2001 Belgian Health Interview Survey tend to be less healthy (i.e. having a higher probability of dying during the follow-up) compared to their counterparts in the general population [32]. This could partly explain why we also found a strong predictive power of the GALI and SRH on mortality in lowly educated participants.

Another shortcoming of the study is that we did not assess the added value of the GALI and SRH for predicting mortality in comparison with more detailed (objective and self-reported) measures of health and functioning. The main objective of the study was to compare the predictive ability of the GALI and SRH on mortality. As the GALI and SRH cover different constructs and given the broad conceptual reach of SRH, we did not include measures of mental and physical health, functional limitations or activities of daily living. Such an adjustment would have affected the GALI and SRH unequally and prevented rigorous comparison between the two global items [19]. Further studies should therefore investigate the added value of the GALI for predicting mortality in the presence of objective and/or subjective measures of functional status.

## Conclusions

This study has shown that the global activity limitation indicator and self-rated health are powerful predictors of mortality in Belgium in different age and socioeconomic groups, among men and among women and over time. The results suggest that the two single-item instruments are useful and complementary measures for assessing the health and functional status of adults in health and non-health surveys.
